# French Adaptation of the Brief Irritability Test: Factor Structure, Psychometric Properties, and Relationship with Depressive Symptoms

**DOI:** 10.5334/pb.1070

**Published:** 2022-01-27

**Authors:** Nellia Bellaert, Wivine Blekic, Kendra G. Kandana Arachchige, Laurent Lefebvre, Mandy Rossignol

**Affiliations:** 1 Department of Cognitive Psychology and Neuropsychology, University of Mons, Mons, BE; 2 Interdisciplinary Research Center in Psychophysiology and Cognitive Electrophysiology, Mons, BE

**Keywords:** Irritability, Factor analysis, Depression, Self-report questionnaire, Scale development, Translation

## Abstract

The Brief Irritability Test (BITe, Holtzman et al., 2014) is a brief, reliable, and valid self-report measure of irritability. Despite the growing interest to understand the underlying causes and consequences of irritability, this questionnaire has not been developed and validated for a French-speaking population yet. In the present study, 413 participants completed our French adaptation of the BITe (i.e., TCI; Test Court d’Irritabilité) and measures of associated constructs (depression, anger, hostility, and aggression) and well-being (life satisfaction and social support). Descriptive, psychometric (i.e., Cronbach alpha and Spearman correlation coefficients), and factor analyses were conducted. An exploratory factor analysis in sample 1 (n = 209), yielded one single factor. The confirmatory factor analysis in sample 2 (n = 204) showed a reasonable fit of this single factor model explaining 55.5% of the variance and presenting a strong internal consistency (α = .80). Compared to the original English questionnaire, the TCI shares similar unidimensional factor organization and correlations with other constructs, although a gender bias was identified, with women scoring higher than men. Irritability was higher among respondents in the age range 17–25, compared to older adults. A hierarchical regression analysis showed that TCI scores significantly predict depressive symptoms when demographics were controlled for. In summary, the TCI presents good psychometric properties and could constitute a valuable tool to evaluate irritability in clinical and research contexts.

Irritability can be defined as an increased sensitivity to negative emotional stimuli leading to an affective response of anger and frustration, and sometimes a behavioral response of aggression (Leibenluft & Stoddard, 2013; Toohey & Di Giuseppe, 2017). Irritability can occur for several reasons including life stress, lack of sleep, pain, or low blood sugar levels, and is therefore very common in the general population throughout the lifespan. Moreover, irritability appears to be a transdiagnostic phenomenon observed in both *externalizing* disorders (e.g., antisocial personality disorder, conduct disorders, substance use disorders, attention-deficit/hyperactivity disorder) and *internalizing* disorders (e.g., unipolar depression, generalized anxiety disorder, posttraumatic stress disorder) (American Psychiatric Association, 2013). Specifically, robust associations between irritability and depression have been established (Savage et al., 2015; Stringaris et al., 2012b). More than half of the adult patients presenting a major depressive disorder experience irritability (Judd et al., 2013) and this symptom has been associated with a greater severity and duration of the depressive episode, a higher risk of suicide (Perlis et al., 2009), a greater psychosocial impairment, and lower levels of life satisfaction (Fava et al., 2010; Judd et al., 2013; Perlis et al., 2005). In young adults with depressive symptoms, the presence of irritability has also been associated with an increased likelihood of engaging in risky behaviors (Pedrelli et al., 2013). Longitudinal studies have shown that the presence of chronic severe irritability (i.e., a construct conceptualized by two clinical manifestations; 1) persistently angry, grumpy, or grouchy mood and 2) behavioral temper outbursts) in children and adolescents robustly predicts later depression, anxiety disorders, suicidality as well as lower educational level and income (Copeland, Brotman & Costello, 2015; Pickles et al., 2010; Stringaris et al., 2009; Vidal-Ribas et al., 2016). Taken together, these poor mental health outcomes emphasize the societal importance of studying the factors causing the emergence and recurrence of irritability in order to improve its identification, prevention, and treatment. Despite the relevance of this concept, the underlying mechanisms of irritability are still poorly understood. Given that it can be measured dimensionally across diagnoses and studied across multiple levels of analysis (i.e., genes, physiology, behavior), irritability fits well within the National Institute of Mental Health (NIMH) Research Domain Criteria (RDoC; Insel et al., 2010) framework. Irritability in youth has been conceptualized as an expression of *frustrative non-reward*, defined as the reactions elicited in response to withdrawal/prevention of expected reward or blocked goal attainment, within the RDoC negative valence domain (Leibenluft, 2011; Vidal-Ribas et al., 2016). A recent pathophysiological model using the RDoC approach (Brotman et al., 2017) suggests that core deficits causing pediatric irritability include aberrant responses to frustration and aberrant approach responses to threat. Regrettably, this model has not been tested in adults while many psychological disturbances and psychiatric disorders are associated with irritability features. The first step in conducting such research is to identify reliable and valid tools to measure irritability.

Irritability has been inconsistently measured in the scientific literature, mainly due to the lack of clarity in the conceptualization of this construct (Toohey & DiGiuseppe, 2017). To the best of our knowledge, there are 11 different questionnaires assessing irritability, which are summarized in ***Table 1***. Recently, the development of irritability scales has significantly increased; six new measures of irritability have been published in the last decade, demonstrating the renewed scientific interest in this construct.

**Table 1:** Characteristics of existing irritability questionnaires.


QUESTIONNAIRE	NUMBER OF ITEMS	REPORTING SOURCE	POPULATION	STATE OR TRAIT IRRITABILITY	STRUCTURE OF IRRITABILITY

Buss-Durkee Hostility Inventory Irritability Subscale – Buss & Durkee, 1957	11	Self-report	Adults	Trait	Unidimensional

Children’s Hostility Inventory Irritability Subscale – Kazdin et al., 1987	6	Parent- and teacher-report	Children	Trait	Unidimensional

Irritability, Depression, Anxiety Scale (IDA) – Snaith et al., 1978	8	Self-report	Adult patients suffering from affective disorders	State (past 2 days)	Bidimensional (i.e., Inwardly or Outwardly directed)

Caprara Irritability Scale (CIS) – Caprara et al., 1985	30	Self-report	Adults	Trait	Unidimensional

Burns Irritability Apathy Scale – Burns et al., 1990	5	Patients’ relative-report	Patients suffering from Huntington or Alzheimer disease	State (since the onset of the illness)	Unidimensional

Irritability Questionnaire (IRQ) – Craig et al., 2008	21	Self- and carer-report	Patients suffering from affective disorders, Huntington, or Alzheimer disease	State (past 2 weeks)	Multidimensional (i.e., Emotional, Cognitive, and Behavioral)

Born Steiner Irritability Scale (BSIS) – Born et al., 2008	14	Self- and observer- report	Women suffering from female-specific mood disorders (i.e., premenstrual, perinatal, and perimenopausal mood disorders)	State (past week)	Unidimensional

I-Epi – Piazzini et al., 2011	18	Self-report	Adult patients suffering from epilepsy	State	Multidimensional (i.e., Physical, Verbal, Temperamental, and Epilepsy functioning)

Affective Reactivity Index (ARI) – Stringaris et al., 2012a	7	Self- and parent-report	Children and adolescents (clinical population and community) Preschoolers (Sugaya et al., 2021)	State (past 6 months)	Unidimensional

Brief Irritability Test (BITe) – Holtzman et al., 2015	5	Self-report	Adults (clinical population and community)	State	Unidimensional

The Cranky Thermometers – Melvin et al., 2018	2	Self-report	Adolescents (clinical population and community)	State (current and past two weeks)	Unidimensional


There are several limitations to these current measurement tools, the first one concerning the definition of the concept of irritability. On the one hand, most questionnaires consider irritability as a mood state (Born et al., 2008; Burns et al., 1990; Craig et al., 2008; Melvin et al., 2018; Snaith et al., 1978; Stringaris et al., 2012b; Piazzini et al., 2011). According to the American Psychological Association (2020), moods are temporary affective states that may last for hours, days, or even weeks, at a low intensity and that do not have a clear object, contrarily to emotions (e.g., anger) which have recognizable antecedents (e.g., an insult), are more intense, and shorter in duration (Kassinove & Tafrate, 2002). In that sense, irritability has been conceptualized as a mood because it can occur “without any “justifiable” triggers and … without any clear antecedents” (Toohey & DiGiuseppe, 2017, p. 96). Also, it can be temporarily induced by stress, physiological/biological factors such as hunger, fatigue, menstruation, pain (Toohey, 2020; Toohey & DiGiuseppe, 2017), or nicotine abstinence (al’Absi et al. 2002) and it is a fluctuating symptom in multiple psychiatric and medical conditions (American Psychiatry Association, 2013). On the other hand, some measures conceptualize irritability as a stable personality trait (Buss & Durkee, 1957; Caprara, 1985; Kazdin et al., 1987), since irritability is defined in the Big Five personality model as a core feature of neuroticism (Costa & McCrae, 1992). As any personality trait, irritability is supposed to show temporal stability and heritability. Research confirms that irritability has shown substantial longitudinal stability (Leadbeater & Homel, 2015; Leibenluft et al., 2006; Moore et al., 2019; Vidal-Ribas et al., 2016), with a tendency to decrease with age (Copeland, Brotman & Costello, 2015). The estimated heritability of irritability was 31% in adolescents (Stringaris et al., 2012b), 37% in male adults (Coccaro et al., 1997), and 54% for tonic irritability (i.e., persistently angry, bad-tempered, or grumpy mood) in youths (Moore et al., 2019). These outcomes suggest that both environmental and genetic factors contribute to the etiology of irritability. Therefore, it is clear that irritability has both state and trait properties, as anger and anxiety (Spielberger et al., 1999). Second, these questionnaires present an overlap between irritability and related constructs, especially with those of anger, aggression, and hostility. Indeed, some items of these scales seem to measure constructs such as verbal aggression (e.g., “*I have yelled at others”*; BSIS, Born et al., 2008), physical aggression (e.g., “*He/she has gotten so angry that he/she has broken things*”; IRQ, Craig et al., 2008), or anger (e.g., “*I have been feeling mad*”; BSIS, Born et al., 2008; “[My child is] *angry most of the time*”, ARI, Stringaris et al., 2012a). Although irritability might be a precursor for anger and aggression (DiGiuseppe & Tafrate, 2007), distinguishing these concepts and their measurement is essential. In contrast to irritability that can be conceptualized either as a mood or a trait, anger is an emotion provoked by a trigger that is more intense and shorter in duration and associated with specific cognitions and changes in physiology (e.g., facial expression) (Kassinove & Tafrate, 2002). Aggression, meanwhile, is defined as any *behavior* directed toward another individual carried out with the intent to harm (Anderson & Bushman, 2002). Third, there is a current lack of consensus on the structure of irritability. For example, the IRQ (Craig et al., 2008) or the IDA (Snaith et al., 1987) have been specially designed to capture the multidimensional nature of irritability. According to Craig et al. (2008), irritability is a mood that predisposes toward certain emotions (e.g., anger), cognitions (e.g., hostile appraisal), and actions (e.g., aggression). Snaith et al. (1987) argue that irritability is expressed either outwardly or inwardly. Other questionnaires (Born et al., 2008; Burns et al., 1990; Buss & Durkee, 1957) support the idea that irritability may be an unidimensional construct. Regrettably, these authors (Born et al., 2008; Burns et al., 1990; Buss & Durkee, 1957; Craig et al., 2008; Snaith et al., 1987) have not conducted factor analyses to test for potential subscales. However, other scale development studies (Caprara et al., 1985; Kazdin et al., 1987; Stringaris et al., 2012a) have found statistical evidence for a single dominant factor, suggesting that all items represent a latent structure of irritability. For example, using principal component analysis, Caprara et al. (1985) demonstrated an unidimensional factorial structure of the CIS, composed of affective (e.g., “*I am often in a bad mood*”, “*I often feel like a powder keg ready to explode*”), cognitive (e.g., “*It is others who provoke my aggression*”, “*When I am right, I am right*”), and behavioral (e.g., “*Sometimes I shout, hit and kick and let off steam*”, “*I can’t help being a little rude to people I don’t like*”) items.

In order to overcome these limitations, the Brief Irritability Test (BITe) has been recently developed (Holtzman et al., 2014). The authors have chosen to conceptualize irritability as a state, consistent with the arguments cited above. The BITe is a self-report measure comprised of five items, rated on a six-point Likert scale (1 = never, 2 = rarely, 3 = sometimes, 4 = often, 5 = very often, 6 = always), designed to assess irritability within the last two weeks. A total score depicting the level of irritability of a subject is calculated by summing the score of each item. In their study, the authors selected 63 candidate items. Twenty-eight of these items were original and developed specially for this study and the other 35 items were extracted from the 21-item IRQ (Craig et al., 2008) and the 14-item BSIS (Born et al., 2008). This irritability item pool as well as standardized measures of associated constructs (i.e., depression, anger, aggression, hostility, and neuroticism) and well-being (i.e., life satisfaction and perceived social support) were completed by 1116 Canadian subjects (877 university students and 229 chronic pain outpatients). Item Response Theory (Embretson & Reise, 2000; Streiner, 2010) was used to determine the five best items to measure the concept of irritability. The results suggested that irritability, as measured by the BITe, might be composed of a unique dominant factor. Indeed, the authors only found weak evidence for smaller item pools that might suggest the importance of subscales. Statistical analyses showed that including any additional items (either the original items or those from the IRQ or the BSIS) did not result in any significant enhancement of the psychometric properties of the BITe. Any added item was either irrelevant or redundant compared to the five items that were finally selected to create the BITe. The 5-item scale showed an excellent internal consistency (α = 0.88). In the validation study, the BITe demonstrated negative correlations with perceived social support (*r* = –.32) and life satisfaction (*r* = –.45). In contrast, moderate to strong correlations have been established between the BITe and depression (*r* = .67), anger (*r* = .51), hostility (*r* = .52), and neuroticism (*r* = .58). The BITe correlated weakly with verbal and physical aggression (*r* = .24 and *r* = .25, respectively). The results showed a little conceptual overlap with these associated constructs. Therefore, this scale allows a greater specificity of the measurement of irritability. Moreover, the BITe has been specifically developed to be suitable for both men and women. Indeed, statistical analyses showed an absence of gender bias on each of the five items and only negligible gender bias on the total test score. In sum, the BITe was presented as a brief, valid, reliable, and unidimensional measure of irritability. The unidimensional structure of irritability has also been confirmed by Deveney et al. (2019) who conducted exploratory bifactor analysis on all items of BITe, ARI, and CIS in a community sample of 458 adults (19–74 years). They found that a dominant factor of irritability explained 40% of response variance on all items.

Even though the BITe appears to be the best irritability scale given that it was built to solve the several limitations cited above (Toohey & DiGiuseppe, 2017), a French version of this questionnaire has not yet been developed and validated. The present study aimed to validate a French adaptation of the BITe (i.e., TCI; *Test Court d’Irritabilité*). In order to fulfill this aim, we first conducted exploratory and confirmatory factor analyses on the five TCI items. The confirmatory factor analysis (CFA) was used to investigate construct validity. Then, we examined convergent and discriminant validity with Pearson’s correlations and regression analysis between irritability and other associated measures. According to the original scale development study, we expected (1) a single dominant factor for the TCI, (2) positive correlations with anger, aggressivity, and hostility, demonstrating a good convergent validity, and (3) negative correlations with perceived social support and life satisfaction, which would demonstrate a good discriminant validity. Additionally, effects of gender and age on irritability levels of the TCI were explored. We here expected (4) a negligible gender bias, and (5) a decrease of irritability levels with age, according to previous longitudinal findings (Copeland, Brotman & Costello, 2015). Finally, given the robust association between irritability and depression in the literature, a regression analysis was conducted to assess convergent validity. We hypothesized (6) a specific association between irritability and depressive symptoms.

## Materials and methods

### Procedures

Participants were recruited through advertisement on social networks (e.g., Facebook groups of the University of Mons and of the city of Mons) during January and February 2020. All participants gave their consent before completing the survey and data anonymity was guaranteed. No compensation was provided for their participation. Ethical approval was provided by the Ethics Committee of the University of Mons. Subjects were first asked about their age, gender, education level, and professional status, before completing an online version of the questionnaires described below.

### Population

In total, 538 French-speaking Belgian participants completed the survey. Participants were screened for exclusion criteria: non-native French speaker, past or current drug/alcohol dependence, medical, or psychiatric condition. As a result, a total of 121 subjects were excluded. The Median Absolute Deviation (MAD) method (Leys et al., 2013) was used to detect outliers on the TCI scores. Using a conservative outlier detection (±3 MAD), 4 participants were found to be outliers because their total scores on the TCI were ≥26. Therefore, the final sample constituted of 413 participants (312 women, 101 men) with an average of 28.31 years of age (*SD* = 12.81). More than half of the subjects (55.4%) were students. The global characteristics of the sample are presented in ***Table 2***.

**Table 2:** Socio-demographic characteristics of the sample.


CHARACTERISTICS		*M(SD)*, N, OR %

Age		28.31 (12.81)

Gender (Female/Male)		312/101

Level of education	Primary school	0.7%

	High school	24.9%

	Bachelor	43.8%

	Master	29.5%

	Ph.D.	1.1%

Professional status	Student	55.4%

	Worker	41.2%

	Unemployed	2.7%

	Retired	0.7%


### Questionnaires

*Brief Irritability Test*. The TCI was developed by two independent Belgian bilingual adults: (a) one French-English bilingual translated the 5 items of the original BITe into French; (b) a second French-English bilingual translated the French version back into English, and discrepancies between the original BITe and the back-translations were discussed between the two translators until a satisfactory solution was found. Beforehand, we received permission from the original first author of the BITe to translate it into French. The French version is presented in Appendix 1.

In order to ease the comparison between the English and French versions of the BITe, we attempted to use French equivalents versions of the measures of associated constructs (i.e., depression, anger, hostility, aggression, life satisfaction, and social support) used in the original published article (Holtzman et al., 2014). [We hypothesized (1) positive correlations with anger, aggressivity, hostility, and depression and (2) negative correlations with perceived social support and life satisfaction.]. We used the French versions of the Beck Depression Inventory (Collet & Cottraux, 1986), the Buss-Perry Aggression Questionnaire (Genoud & Zimmermman, 2009), the “anger-hostility” subscale from the NEO-PI-R (Costa, McCrae & Rolland, 1998, 2003), and the Satisfaction with Life Scale (Blais et al., 1989). We replaced the State-Trait Anger Expression Inventory–2 (Spielberger, 1999), copyrighted by Psychological Assessment Resources, Inc., by a copyright-free French translation of the Clinical Anger Scale (Goulet, n.d.). To assess social support, we used the French version of the Social Support Questionnaire (Bruchon-Schweitzer et al., 2003), instead of the Interpersonal Support Evaluation List–12 (Cohen & Hoberman, 1983) because the latter has not been validated in French. The questionnaires used for the current study are described below.

*Beck Depression Inventory – Short Form* (BDI; Beck et al., 1961). We used the 13-item French version validated by Pichot & Lempérière (1964), which have strong internal consistency (α = .90) and adequate test-rest reliability (*r* = .62, *p* < .001) over a 4-month period (Bourque & Beaudette, 1982). It aims to measure the presence of depressive symptoms. Each item is subdivided into 4 statements, rated from 0 to 3, and depicts a depressive symptom. Each statement corresponds to the severity of this symptom. Depression is considered suspected from 4 to 7 points, moderate from 8 to 15 points, and severe from 16 points.

*Clinical Anger Scale* (CAS; Snell et al., 1995). The CAS is a 21-item self-report scale measuring the presence of clinical anger symptoms. Each item is subdivided into 4 statements, rated from 0 to 3. Each item depicts an anger symptom and each statement corresponds to the severity of this symptom. The clinical anger level of the respondent is considered as minimal from 0 to 13 points, mild from 14 to 19 points, moderate from 20 to 28 points, and severe from 29 to 63 points. It has been translated in French by Goulet (n.d.) but has not been validated yet.

*NEO PI-R: Anger Subscale* (Costa & McCrae, 1992). The subscale « anger-hostility » from the NEO PI-R Inventory is a single factor 8-item scale measuring general feelings of anger and is part of the second facet of neuroticism. Items are rated on a 5-point Likert scale (1 = *strongly disagree*, 5 = *strongly agree*). The subscale has been validated in French by Costa, McCrae & Rolland (1998, 2003). The French version demonstrated good internal consistency in a student sample (α = .70) and a military sample (α = .64) (Rolland, Parker & Stumpf, 1998).

*Buss-Perry Aggression Questionnaire – Short Form* (AQ; Buss & Perry, 1992). It is a 29-item self-report measure of aggression comprised of 4 subscales: verbal aggression, physical aggression, anger, and hostility. Items are rated on a 6-point Likert scale from “*Not at all like me*” to “*Completely like me*”. A 12-item version has been proposed by Bryant and Smith (2001) and validated in French by Genoud & Zimmermman (2009). The internal consistency coefficients of the 12-item French version were good for the total scale (α = .80), acceptable for the physical aggression (α = .72) and anger (α = .72) subscales, and questionable for the verbal aggression (α = .61) and hostility (α = .61) subscale.

*Satisfaction With Life Scale* (SWLS; Diener et al., 1985). This scale, that proposes five items rated on a 7-point Likert scale (1 = *strongly disagree* to 7 = *strongly agree*), is the most widely used measure of life satisfaction and has strong psychometric properties (Pavot & Deiner, 1993). We used the French version validated by Blais et al. (1989) that showed an excellent internal consistency (α = .87) and an adequate test-retest reliability (*r* = .64, *p* < .001).

*Social support questionnaire* (SSQ; Sarason et al., 1987). It is an economic 6-item scale that measures two dimensions of perceived social support: availability and satisfaction. For each of the six items, respondents indicate the number of people (maximum 9) available to provide support in each situation described. Then, they rate their overall level of satisfaction (from 1 to 6) with the support given in each of the situations. Two total scores are calculated, one for availability and one for satisfaction. The SSQ has excellent psychometric properties and has been adapted in French (Bruchon-Schweitzer et al., 2003). The French SSQ demonstrated an internal consistency of 0.86 for availability and 0.87 for satisfaction, as well as excellent test-retest reliability (0.89 for availability and 0.84 for satisfaction).

### Statistical analysis

SPSS 25 was used for all statistical analyses, except for the confirmatory factor analysis that was performed on RStudio. For the exploratory factor analysis, the Kaiser–Meyer–Olkin measure and the Bartlett measure indicated that the data were suitable for factor analysis. The number of components to be extracted was identified by a scree plot and Horn’s parallel analysis. Exploratory factor analysis was conducted with oblique (Direct Oblimin) rotation and Kaiser normalization. Confirmatory factor analysis was computed with the Lavaan package in RStudio (Rosseel, 2012). We used four fit indices to evaluate the overall fit of the tested model. The fit indices used were the Confirmatory Fit index (CFI) and the Tucker–Lewis Index (TLI), where values of .95 or higher were considered indicative of a good fitting model (Bentler, 1990). We also used the Root Mean Squared Error of Approximation (RMSEA) and the Standardized Root Mean Residual (SRMR), with values of .06 or under as indicative of good fit (Bentler, 1995; Browne & Cudeck, 1993). The chi-square value was not interpreted because it is sensitive to sample size and is likely to be significant in large samples regardless of model fit (Kline, 2011). Correlations were calculated between TCI and measures of associated constructs using Pearson coefficient correlation. Because individual items of the TCI were not normally distributed in our sample, we used Mann-Whitney U tests to assess gender and age differences. Finally, given the strong correlation between the variables of interest, we conducted a hierarchical regression analysis to examine the relationship between the TCI and depressive symptoms. The anonymized dataset can be downloaded on the Open Science Framework on the following link: *https://osf.io/vs8gr/*.

## Results

**Figure 1:**
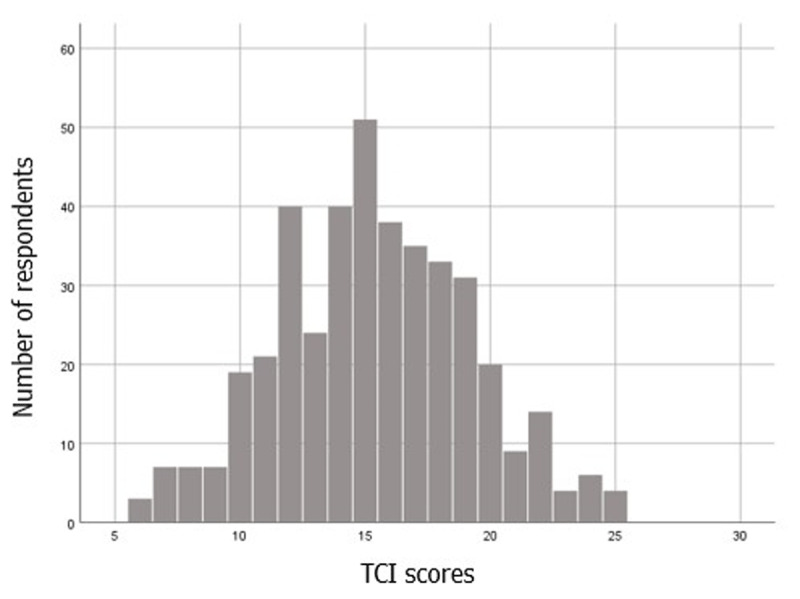
The TCI scores of respondents displayed as a histogram. *Note*: TCI = Test Court d’Irritabilité (French Brief Irritability Test).

The distribution of TCI scores in our entire sample (*N* = 413) is displayed in ***Figure 1***. The mean on the TCI for our sample was 15.36 (*SD* = 3.87, range 6–26; item summation scoring). The median was 15. We conducted a one-sample *T*-Test and we found that the mean of our study was significantly higher than the mean of Holtzman et al. (2014) (*M* = 12.55), *t*(412) = 14.76, *p* < .001.

A random split sample procedure was performed in the total sample, yielding a first sample of 209 subjects and a second sample of 204 subjects. The total score on the TCI in sample 1 (*M* = 15.22; *SD* = 2.83) and sample 2 (*M* = 15.50; *SD* = 3.91) did not differ significantly (*U* = 20 971, *p* = .774).

### Factor analyses

#### Exploratory Factor Analysis

The data collected were suitable for factor analysis (KMO = .808, Bartlett = .000). Both the scree plot and the Horn’s parallel analysis suggested the extraction of a single factor. The results can be seen in ***Table 3***. The single factor model explained 55.5% of the variance and had a strong internal consistency (a = .80). Item 5 “I have been feeling irritable” had the largest factor loadings (.81) on the single factor.

**Table 3:** Rotated factor loadings from the exploratory (EFA) and confirmatory (CFA) factor analyses of the TCI items in two split samples.


DESCRIPTION OF ITEM CONTENT		EFA RESULTS IN SAMPLE 1 (*n* = 209)	CFA RESULTS IN SAMPLE 2 (*n* = 204)

		FACTOR « IRRITABILITY »	

5.	Je me sens irritable	.81	.86

1.	J’ai été grincheux	.75	.78

3.	D’autres personnes m’énervent	.80	.62

4.	Des choses me dérangent plus qu’elles le font d’habitude	.78	.60

2.	J’ai eu l’impression que j’allais craquer	.55	.60


*Note*: EFA = Exploratory Factor Analysis; CFA = Confirmatory Factor Analysis; TCI = Test Court d’Irritabilité (French Brief Irritability Test).

#### Confirmatory Factor Analysis

The single factor model that emerged in sample 1 was evaluated with a Confirmatory Factor Analysis in sample 2 and fitted reasonably well (RMSEA = .096; SRMR = .035; CFI = .973; TLI = .95) although the RMSEA value fell above the .06 criteria (Browne & Cudeck, 1993). The standardized factor loadings in this model are indicated in ***Table 3***. All factor loadings are in an acceptable range (λ = .60–.86).

### Relationships between irritability and associated constructs

#### Preliminary analysis

Means, standard deviations and internal consistency correlations for the TCI and questionnaires of associated constructs are represented in ***Table 4***. All questionnaires demonstrated internal consistency ranging from acceptable to good (Cronbach’s alphas ≥ .71), except for the verbal aggression subscale of the Aggression Questionnaire where it was unacceptable (a = .45). Given the low reliability of the verbal aggression subscale, we will not proceed with any further analyses with this scale.

**Table 4:** Sample size, internal consistency coefficients, means, standard deviations for the TCI and measures of associated constructs.


QUESTIONNAIRES	*N*	α	*M*	*SD*

TCI	413	.80	15.36	3.87

BDI-13	413	.81	5.96	4.78

CAS	413	.84	8.17	6.21

NEO PI-R : anger subscale	413	.73	22.98	5.11

AQ	413	.76	25.15	7.32

Physical Aggression	413	.73	4.98	2.54

Verbal Aggression	413	.45	6.92	2.26

Hostility	413	.71	6.23	3.02

Anger	413	.71	7.01	2.93

SWLS	413	.86	24.34	6.47

SSQ – Availability	413	.88	25.93	11.58

SSQ – Satisfaction	413	.91	29.20	5.61


*Note*: TCI = *Test Court d’Irritabilité* (French Brief Irritability Test); BDI-13 = Beck Depression Inventory 13 items; CAS = Clinical Anger Scale; NEO PI-R = Revised NEO Personality Inventory; AQ = Aggression Questionnaire; SWLS = Satisfaction with Life Scale; SSQ = Social Support Questionnaire.

#### Correlations

Pearson correlations were calculated between TCI and associated constructs (depression, anger, hostility, etc.). These correlations are presented in ***Table 5***.

**Table 5:** Correlations between TCI and associated constructs.


	TCI	*P* VALUE

BDI-13	.56	<.001

CAS	.59	<.001

NEO PI-R – Anger subscale	.54	<.001

AQ total	.45	<.001

AQ (Physical aggression)	.08	0.123

AQ (Hostility)	.40	<.001

AQ (Anger)	.49	<.001

SWLS	–.39	<.001

SSQ – Availability	–.31	<.001

SSQ – Satisfaction	–.23	<.001


*Note*: TCI = *Test Court d’Irritabilité* (French Brief Irritability Test); BDI-13 = Beck Depression Inventory 13 items; CAS = Clinical Anger Scale; NEO PI-R = Revised NEO Personality Inventory; AQ = Aggression Questionnaire; SWLS = Satisfaction with Life Scale; SSQ = Social Support Questionnaire.

TCI score was positively correlated with measures of depression, anger, and hostility (all correlations *p* < .001). In contrast, TCI score was negatively correlated with measures of life satisfaction and perceived social support (all correlations *p* < .001). TCI score was not correlated with physical aggression (correlation *p* = .123).

#### Gender, age, and professional status effects

The Kolmogorov-Smirnov test revealed that the items of TCI and other questionnaires were not normally distributed in our sample. Therefore, non-parametric Mann-Whitney U tests were conducted. A main effect of gender (*U* = 10 810, *p* < .001) showed that women scored higher (*M* = 15.85; *SD* = 3.81) than men (*M* = 13.84; *SD* = 3.67) on irritability. Significant gender differences were also found for measures of depressive symptoms (*U* = 12 626, *p* < .005), with higher depressive symptoms among women (*M* = 6.26; *SD* = 4.76) than men (*M* = 5.02; *SD* = 4.74). The anger-hostility subscale of the NEO PI-R (*U* = 13 259, *p* = .016) showed a gender difference with women (*M* = 23.30; *SD* = 5.29) scoring higher than men (*M* = 22; *SD* = 4.37). No gender differences were found for measures of life satisfaction (*U* = 15 703, *p* = .960), anger on the CAS (*U* = 13 761, *p* = .055), and the two dimensions of social support (availability: *U* = 15 469, *p* = .783; satisfaction: *U* = 13 557, *p* = .054). Regarding the AQ, the total score (*U* = 14 361, *p* = .181), anger subscale (*U* = 13 864, *p* = .068), and hostility subscale (*U* = 15 123, *p* = .54) showed no effect of gender. However, the physical aggression subscale (*U* = 10 212, *p* < .001) was significatively higher among men (*M* = 6.23; *SD* = 3.05), than women (*M* = 4.58; *SD* = 2.21).

A Kruskal-Wallis test showed an effect of age groups on the TCI scores, *H*(2) = 30.47, *p* < .001. Post-hoc Mann-Whitney U tests were used to compare all pairs of groups. Irritability scores of participants aged from 17 to 25 years old (*M* = 16.06; *SD* = 3.84) were significatively higher than those of participants aged from 26 to 55 years old (*M* = 14.15; *SD* = 3.33; *U* = 11 391, *p* < .001), and older than 55 years (*M* = 12; *SD* = 3.9; *U* = 1235, *p* < .001). The difference between the last two groups was also statistically significant (*U* = 699, *p* < .005). TCI scores were also significatively higher among students (*M* = 16.21; *SD* = 3.76) than among workers (*M* = 14.35; *SD* = 3.75; *U* = 14 242, *p* < .001). The relation between these variables (age and professional status) was significant (χ^2^ (4) = 242,438, *p* < .001). Logically, young adults were more likely to be students.

#### Relationship with depression

TCI scores were significantly correlated to BDI scores (*r* = .56, *p* < .001), indicating that symptoms of depression are associated with higher irritability rates. A hierarchical regression analysis was conducted, and the TCI scale was used to predict depression scores. Gender was entered on the first step; age was entered on the second step and education level was entered on the third step. The TCI scale was then entered on the fourth step. The results are shown in ***Table 6***. In the prediction of depression scores, only the TCI scale makes a significant contribution when demographics (i.e., gender, age, and education level) have been taken into account, *R*^2^ = .312, *F*(1) = 186.13, *p* < .001. This indicates a unique relationship between irritability and depression. We suppose that this association might be bidirectional. However, our cross-sectional design prevents us from testing this hypothesis as it was not the main goal of the current study.

**Table 6:** Results from the hierarchical regression analysis using the TCI to predict depression scores (N = 413).


	INDEX			

DEPENDENT VARIABLE: DEPRESSION (BDI 13)	B	SE B	β	T

Step 1 (R^2^ = .016)				

Gender			–.014	–.328

Step 2 (R^2^ = .035)				

Age			–.030	–.703

Step 3 (R^2^ = .046)				

Education			–.039	–.941

Step 4 (R^2^ = .312)				

TCI	.69	.051	.558	13.643*


*Note*: TCI = *Test Court d’Irritabilité* (French Brief Irritability Test); BDI-13 = Beck Depression Inventory 13 items. * *p* < .001.

## Discussion

The present study aimed to develop and validate a French version of the Brief Irritability Test (i.e., TCI; *Test Court d’Irritabilité*) and to examine the similarities with the original English version. First, we found higher irritability rates in our study compared to the mean of the original version of Holtzman et al. (2014) (15.36 vs. 12.55, respectively). Our sample was mostly constituted of students (55.4%) and female participants (75.5%). These two specific populations have been demonstrated to manifest higher irritability rate (Fava et al., 2010; Perlis et al., 2009; Piazzini et al., 2011) and therefore, it may have risen the irritability rate of our sample. The higher irritability mean in our study might also be attributed to cultural differences in the way Canadian and Belgian populations experience and manifest feelings of irritability, as emotional experiences are influenced by the cultural environment (Lim, 2016). In fact, a preliminary study reported slight differences in the experience of irritability across countries (e.g., China, USA, Ireland, UK, etc.) (Toohey, 2020). However, that study included only English-speaking participants and it is still unknown how languages and cultures modify the way people describe, perceive, and experience feelings of irritability. This deserves further examination in the future.

Second, results supported a single dominant factor of the TCI as hypothesized, demonstrating the good construct validity of our instrument. Therefore, the TCI and the original BITe appeared to have a similar factor organization. Consistently with previous studies, our findings demonstrated that the construct of irritability can be effectively captured using only five items, that represent increased sensitivity to provocation and temper loss (Deveney et al., 2019; Holtzman et al., 2014).

Third, we found strong correlations between measures of irritability and both hostility and anger (*r*s between .40 and .59). Hostility is defined as a complex of negative attitudes, beliefs, and appraisals concerning others (Smith, 1992). It incorporates cognitive traits such as suspiciousness, resentment, and distrust of others (Evans et al., 1999). Defined this way, hostility is not synonym to irritability, but they can co-occur. Craig et al. (2008) stated that anger is an *emotion* associated with irritable *mood*. Thus, they differ by their nature. Emotions last for seconds or minutes, have unique facial expression and recognizable antecedents. In contrast, moods have longer duration, may not have specific facial expression and do not necessarily occur in relation to an external object (Craig et al., 2008). According to the definition of Toohey & DiGiuseppe (2017), anger is considered as a consequence of irritability, which can explain their strong correlation in the present study. Therefore, they cannot be used as interchangeable terms because anger can occur in the absence of irritability. Furthermore, scores on the TCI did not correlate with physical aggression, emphasizing the need to distinguish irritability from aggression (Toohey & DiGiuseppe, 2017). Aggression can be considered as a behavioral consequence of irritability, given that irritability decreases the threshold for exaggerated responses to frustrating stimuli or events (Deveney et al., 2013; Tseng et al., 2019). However, the negative affective responses associated with irritability (e.g., anger) may not be outwardly, or behaviorally, expressed (Barata et al., 2016), explaining the absence of correlation. To assess physical aggression, we used the French 12-item version of the AQ while the English 29-item form was used by Holtzman et al. (2014) and it thus may be possible that the two versions of these questionnaires might be differentially associated with irritability measures. Unfortunately, these two versions and their respective associations with irritability have never been compared, which hinders us to pinpoint the exact reason for this negligible correlation. Nonetheless, distinctions between irritability as measured by the BITe and aggression as measured by the 29-item AQ have been found previously (Deveney et al., 2019). The authors have compared similarity between irritability and the AQ’s subscales using Ward’s hierarchical clustering method (see Murtagh & Legendre, 2014 for more details about this method). In a large community sample of adults, irritability clustered with the anger (*r* = 0.83) and hostility (*r* = 0.67) subscales, but the verbal aggression and physical aggression subscales cluster together in a separate group with constructs of reactive and proactive aggression measured by the Reactive and Proactive Aggression Questionnaire (Raine et al., 2006). This provides further empirical evidence that irritability needs to be clearly distinguished from aggression. We were not able to explore the correlation between irritability and verbal aggression, given the insufficient internal consistency of this subscale. Previous studies conducted with the 29-item AQ have also found low Cronbach’s alphas for the verbal aggression subscale in Dutch students (α = .50; Meesters et al., 1996), Dutch adolescent male offenders (α = .51; Morren & Meesters, 2002), and Japanese and Spanish students (α = .64; Ramirez et al., 2001). Unacceptably low reliability scores for the verbal aggression subscale have also been found for the 12-item AQ in an Asian adolescent sample (α = .50; Ang, 2007) and in the French validation study (α = .61; Genoud & Zimmermman, 2009). Taken together, these findings suggest that the verbal aggression subscales from both the AQ short and long forms have a limited internal consistency. The low Cronbach’s alphas estimates in these studies as well as in the present sample may also be possibly explained by the limited number of items per subscale (i.e., 3 items for the short version and 5 items for the long version), language, culture, and age (Ang, 2007).

Scores on the TCI demonstrated inverse correlations with life satisfaction and perceived social support. The correlations between the TCI and associated constructs as well as measures of well-being are somewhat similar to the correlations found in the original English questionnaire; our French version correlated similarly with AQ-anger (*r* = .49 vs. .56 in Holtzman et al., 2014), AQ-hostility (*r* = .40 vs. .52), depression (*r* = .56 vs. .67), social support (*r* = –.31 for availability and *r* = –.23 for satisfaction vs. –.32), and life satisfaction (*r* = –.39 vs. –.45). However, given that this study had a cross-sectional design, no causality can be inferred. As Holtzman et al. (2014), we suspect that these associations are bidirectional. Considering the positive associations with the associated constructs of anger, hostility, and depression, and the negative associations with life satisfaction and social support, we conclude that the French BITe has both good convergent and discriminant validity.

We explored the effect of gender on irritability and found that women reported greater irritability. Our results are in line with previous findings of higher irritability rates among females rather than males in various population: adolescents from general population (Leibenluft et al., 2006), adult patients with epilepsy (Piazzini et al., 2011), college students with depressive symptoms (Pedrelli et al., 2015), and adult patients with major depressive disorder seeking treatment (Marcus et al., 2008; Perlis et al., 2009). However, this is inconsistent with the English original version that was partly developed to avoid gender bias (Holtzman et al., 2014). The presence of a gender bias in our sample may be explained by different factors. First, our female participants were more likely to be younger and we found an impact of age on irritability rate among respondents. In our community sample, irritability was especially higher among participants in the age range 17–25, which are mostly students. Second, our female subsample reported higher depressive symptoms than men and we found that measures of irritability and depression correlated highly in our sample. A significant body of the literature links irritability with depression (Fava et al., 2010; Jha et al., 2020; Judd et al., 2013; Pedrelli et al., 2015; Stringaris et al., 2009; Vidal-Ribas et al., 2016; Vidal-Ribas & Stringaris, 2021), and evidence showed that in a large cohort of MDD patients, irritability was more common among female and younger participants (Perlis et al., 2009). Taken together, these variables (i.e., age, professional status, and depressive symptoms) might account for the emergence of a gender bias in our study. A future study in which these variables are matched between male and female is needed to further analyze the gender bias in the TCI.

Lastly, we examined the association between irritability and depressive symptoms given the high prevalence of irritability among patients with major depressive disorder. We found a strong correlation between irritability and depression (*r* = .56). We also found that TCI scores predicted depressive symptoms when demographics variables were controlled for. In the general population, irritability appears to be associated with more severe depressive symptoms. This finding is consistent with previous observations in college students (Pedrelli et al., 2015), adults from general population (Verhoeven et al., 2011), and adult patients with major depressive disorders (Perlis et al., 2009). However, these studies assessed irritability using a single item (i.e., item 11 of the Beck Depression Inventory was used to assess the presence of irritability within the past week), which generally produce less reliable and valid results than multi-item scales (Bowling, 2005; Liu, 2003). Further research is needed to explore the links between the TCI and disorders in which irritability is a common symptom such as major depressive or generalized anxiety disorders.

This study should be considered in light of some limitations. First, the cross-sectional design of the present study prevents us from inferring temporality and causality between irritability, associated constructs, and measures of well-being, and examining the predictive validity of our scale. A longitudinal study measuring the ability of TCI score to predict the presence and severity of depressive symptoms is needed to test predictive validity. It also limits the ability to examine the stability of the TCI. Second, data of the present study was collected online, similarly to the original development study of the BITe (Holtzman et al., 2014). Thus, clinicians and researchers that will use the paper version of our scale should be aware that the psychometric properties might differ from those of this current study. Even though systematics reviews found that reliability between online and paper versions of self-administered questionnaires is high (Alfonsson, Maathz & Hursti, 2014; Gwaltney, Shields & Shiffman, 2008), the reliability and validity of our online TCI still need to be compared to a paper-administered version in a research laboratory setting. Third, the low reliability of the verbal aggression subscale in our sample prevents us from exploring the association between irritability and this specific subtype of aggression. Fourth, the content of our advertisement on social media may have introduced a recruitment bias. The study was advertised to the participants as follow: “*Our goal is to validate in French a questionnaire measuring the symptom of irritability, which is defined as a proneness to become easily annoyed or angry*”. As a result, it is possible that people who are more prone to experience feelings of irritability, annoyance, and anger were more likely to engage in our study, which presents potential for self-selection bias and might explain the higher irritability rates in our study compared to Holtzman et al. (2014). Indeed, previous studies using different wording and terminology to advertise mental health surveys on Facebook have demonstrated that it can lead to different participation rates and engagement (Batterham, 2014; Choi et al., 2017). Thus, a next step for the validation of the TCI will be to use an advertisement content that do not explicitly mention that the researchers are interested in irritability and to investigate whether the characteristics and the irritability scores of the sample differ from our present study. Lastly, the majority of our sample (i.e., 94.7%) received at least a high school degree and therefore, the understanding of the items may need to be verified in a small-scale pilot study if researchers wish to use this questionnaire in lower educated populations. Future studies should also explore the actual gender and cultural differences in irritability and examine the test-retest reliability of the TCI.

## Conclusion

The TCI appears to have good psychometric properties as the first French self-report scale to measure irritability. Thus, this brief and reliable tool could be used in studies interested in determining the causes, consequences, and treatment of irritability.

## Appendices

**Appendix 1:** Test Court d’Irritabilité. **Consigne**: Veuillez indiquer à quelle fréquence vous vous êtes senti(e) ou vous êtes comporté(e) de la manière suivante, **au cours des deux dernières semaines, y compris aujourd’hui**.

		JAMAIS	RAREMENT	PARFOIS	SOUVENT	TRÈS SOUVENT	TOUJOURS

1.	J’ai été grincheux.						

2.	J’ai eu l’impression que j’allais craquer.						

3.	D’autres personnes m’énervent.						

4.	Des choses me dérangent plus qu’elles le font d’habitude.						

5.	Je me sens irritable.						
